# Plasma Small Extracellular Vesicle Cardiac miRNA Expression in Patients with Ischemic Heart Failure, Randomized to Percutaneous Intramyocardial Treatment of Adipose Derived Stem Cells or Placebo: Subanalysis of the SCIENCE Study

**DOI:** 10.3390/ijms241310647

**Published:** 2023-06-26

**Authors:** Denise Traxler, Varius Dannenberg, Katrin Zlabinger, Alfred Gugerell, Julia Mester-Tonczar, Dominika Lukovic, Andreas Spannbauer, Ena Hasimbegovic, Jens Kastrup, Mariann Gyöngyösi

**Affiliations:** 1 Division of Cardiology, Department of Internal Medicine II and Department of Oral and Maxillofacial Surgery, Medical University of Vienna, 1090 Vienna, Austria; denise.traxler-weidenauer@meduniwien.ac.at; 2 Division of Cardiology, Department of Internal Medicine II, Medical University of Vienna, 1090 Vienna, Austria; 3 Division of Cardiology, Department of Internal Medicine II, Department of Thoracic Surgery, Laboratory for Cardiac and Thoracic Diagnosis and Regeneration, Medical University of Vienna, 1090 Vienna, Austria; 4 Cardiology Stem Cell Centre, Department of Cardiology, Centre for Cardiac, Vascular, Pulmonary and Infectious Diseases, Rigshospitalet, University of Copenhagen, Henrik Harpestrengs Vej 4, 2100 Copenhagen, Denmark

**Keywords:** extracellular vesicles, microRNA, ischemic heart disease, adipose-derived stem cells

## Abstract

Small extracellular vesicles (EVs) and their cargo are an important component of cell-to-cell communication in cardiac disease. Allogeneic adipose derived stem cells (ADSCs) are thought to be a potential approach for cardiac regenerative therapy in ischemic heart disease. The SCIENCE study investigated the effect of ADSCs administered via intramyocardial injection on cardiac function in patients with ischemic heart disease. The aim of this substudy, based on samples from 15 patients, was to explore small EV miRNA dynamics after treatment with ADSCs compared to a placebo. Small EVs were isolated at several timepoints after the percutaneous intramyocardial application of ADSCs. No significant effect of ADSC treatment on small EV concentration was detected. After 12 months, the expression of miR-126 decreased significantly in ADSC patients, but not in the placebo-treated group. However, all cardiac miRNAs correlated with plasma cardiac biomarkers. In line with the overall negative results of the SCIENCE study, with the exception of one miR, we did not detect any significant regulation of small EV miRNAs in this patient collective.

## 1. Introduction

Ischemic heart disease resulted in 17.9 million deaths worldwide in 2019, despite improvements in diagnosis and treatment within recent years. (WHO, 2021) The current treatment approaches comprise pharmaceutical and surgical therapies, which alleviate symptoms and impede revers remodeling [1]. However, after exhausting all therapeutic options, some patients continue to exhibit symptoms due to progression of the disease, and the permanent loss of functional myocardial tissue has not yet been adequately addressed. With an overall progressively aging society and general improvements in medical care, the incidence of post-myocardial infarction heart failure is increasing, and cardiac regeneration therapy remains an evergreen topic in cardiovascular medicine and research. The regenerative capacity of stem cells is thought to offer a promising new treatment approach in various cardiovascular diseases, including ischemic heart disease. Preclinical large animal models have proven stem cell therapy to be both safe and moderately effective. Cell-based cardiac regenerative therapies in chronic heart failure aim to restore myocardial function through either cell-mediated or paracrine mechanisms [2,3]. Adipose-derived stem cells (ADSCs) are a subtype of mesenchymal stem cells (MSCs) which can differentiate into various cell types. In contrast with other MSCs, ADSCs are easily available and abundant, whilst possessing a similar regenerative potential [4]. Their cardioprotective effects are elicited by preventing apoptosis, inducing angiogenesis and suppression of inflammation and fibrosis [5,6,7].

Extracellular vesicles (EVs) are small membrane-enclosed vesicles originating from various cell types. They are found in biological fluids such as blood, urine, and cerebrospinal fluid. In recent years, they have been studied extensively, and their role in cell-to-cell communication is of special interest [8,9]. However, the unclear and overlapping nomenclature of EV subtypes (e.g., exosomes, ectosomes, microvesicles, or apoptotic bodies) poses an obstacle to the evaluation of available research. Isolated EVs below 200 nm in size are considered small EVs (once presumed to be exosomes) [10,11]. Small EVs are double-membraned vesicles formed from endosomes which express distinct surface markers (e.g., CD9, CD63, CD81) [10]. The incorporation of EV content in target cells is a widely accepted mechanism of cell-to-cell communication elicited by EVs. EV uptake is highly selective, and dependent on surface proteins of both EVs and target cells. EV uptake mechanisms include endocytosis and membrane fusion. Transmembrane receptor bound signaling without uptake has also been described [12].

Mature microRNAs (miRNAs) are small non-coding RNAs with a length of 18–25 nucleotides that regulate protein expression by pairing with the 3′ untranslated region (UTR) of target messenger RNAs (mRNA), resulting in either mRNA cleavage or repression of translation [13,14]. In general, miRNAs can bind to a number of mRNAs, which results in a myriad of effects elicited by a single miRNA [15]. Nevertheless, several miRNA clusters exist for specific tissue types or diseases. Thus, miRNAs are important (patho)physiological regulators of the cardiovascular system [16,17]. In cardiac regeneration, several miRNAs have already been extensively investigated. Based on the literature, we chose six miRNAs as the focus of our study (miR-21, miR-24, miR-126, miR-1, miR-133a and miR-499). All six miRNAs were already described to be either systemically expressed in ischemic heart disease, or are known regulators of cardiac regenerations by regulation of angiogenesis and apoptosis. miR-126 is of special interest, as it has already been used in a small animal model as a therapeutic agent incorporated in small EVs [18,19,20,21,22,23]. Circulating miRNAs are either bound to proteins and lipids or transported in EVs, which protects them from degradation [24,25,26]. Mechanisms directing the selection of packed miRNAs in EVs, the type of cells targeted by miRNA packed EVs, and the extent of protein regulation in target cells have not yet been elucidated. The cardiovascular system is comprised of a plethora of different cell types that need to interact in an orderly well-regulated fashion in order to enable smooth physiological functioning of its components. EV mediated miRNA transfer is thought to be a component of this system.

In contrast with small and large animal studies, human cardiac stem cell treatments have comparatively disappointing results [3,27,28,29]. Several approaches, which aimed to account for cell type, source of cell type, or mode of delivery have been assessed in order to improve the efficacy of human cardiac regeneration treatments. The use of allogeneic cells instead of autologous cells is thought to offer greater regenerative potential, as patients in need of cardiac regenerative therapy tend to be older and suffer from several comorbidities, which has deleterious effects on the regenerative capacity of stem cells. Furthermore, autologous cell-based therapies require extensive preparation within a narrow time frame. Moreover, the mode of delivery is a highly debated factor, the most common application route is the delivery via epicardial, intramyocardial or intracoronary injections [30]. The percutaneous intramyocardial injection of regenerative substances offers the advantage of precise delivery of cell or gene-based advanced therapy medical products (ATMP) to the ischemic area [31]. 

The SCIENCE study was a multicenter, randomized, double-blinded, placebo-controlled study that was conducted in six European centers (ClinicalTrial.gov ID: NCT02673164). The study design, as well as the study results, have been published previously. Briefly, the aim of the SCIENCE trial was to investigate the effect of a 3D electroanatomical mapping (NOGA system) guided percutaneous intramyocardial injection with allogeneic ADSCs on cardiac function. ADSCs were derived from healthy donors, and processed and stored in Denmark. Isotonic saline served as the placebo. Intramyocardial treatment with allogeneic ADSCs was proven to be safe, albeit without causing significant improvements in cardiac function or clinical symptoms during the 1-year follow up [32].

The aim of our SCIENCE substudy was to investigate the effect of allogeneic ADSC treatment on small EVs isolated from peripheral blood, and small EV miRNA expression of selected miRNAs in this setting, in order to detect possible changes in the landscape of small EVs induced by percutaneous intramyocardial application of allogeneic ADSCs. 

## 2. Results

### 2.1. Description of the Study Collective

Our center included and randomized 15 patients to receive either the allogeneic ADSCs or the placebo. All 15 patients were male, and the mean age was 62 ± 8 years. Ten patients received the active substance (allogeneic ADSCs) (62 ± 8 years) and 5 patients the placebo (61 ± 9 years), administered by percutaneous 3D NOGA-guided intramyocardial injections. Thirteen patients were previous or active smokers, with a mean of 40.1 ± 21.6 pack years (ADSC: 43.2 ± 24.8 years, placebo: 36.0 ± 15.4 years, *p* = 0.613). 

### 2.2. Treatment-Related Events

No treatment-related adverse events were observed, and the twelve-month follow-up timepoint was reached by all patients.

### 2.3. Comparison of Small Extracellular Vesicle Isolation Methods

Small EV protein, RNA, and particle concentration and size, as well as the particle to protein ratio and particle to RNA ratio, were compared between ultracentrifugation with three different starting volumes and Exoquick. Further microvesicles and plasma were analyzed as well.

**Figure 1:**
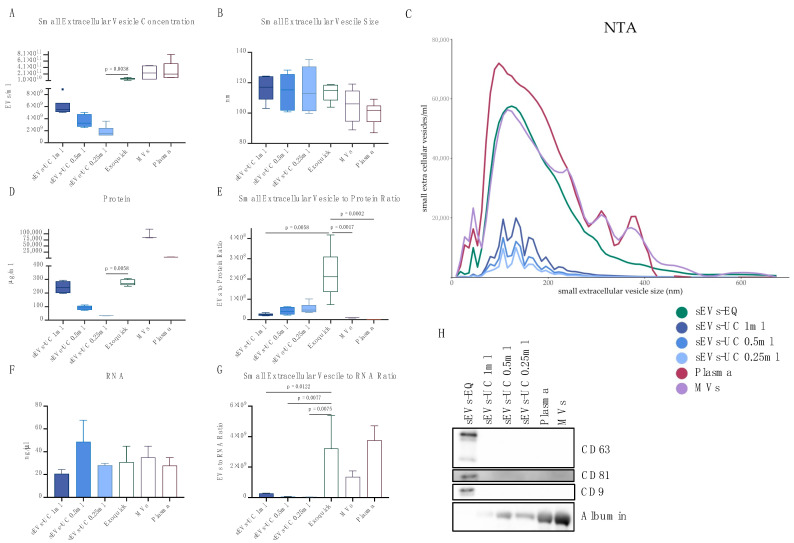
Small EV concentration (**A**), vesicle size (**B**), spectrum of vesicle size (**C**), protein concentration (**D**), vesicle to protein ratio (**E**), RNA concentration (**F**), vesicle to RNA ratio (**G**), and expression of CD63, CD81, CD9 and albumin (**H**) in sEVs-EQ, sEVs-UC 1 mL, sEVs-UC 0.5 mL, sEVs-UC 0.25 mL, microvesicles (MVs) and plasma.

Overall, a small EV isolation with Exoquick performed superiorly in contrast to ultracentrifugation. Small EV concentration in sEVs-EQ was significantly increased in comparison with sEVs-UC 0.25 mL (median [IQR]: 5.93 × 10^10^ [4.08 × 10^10^–8.43 × 10^10^] vs. 1.55 × 10^9^ [1.23 × 10^9^–2.44 × 10^9^] particles/mL; *p* = 0.0038, Figure 1A). Small EV size was not significantly altered by the isolation method (Figure 1B). The exact NTA spectrum is depicted in Figure 1C. However, the protein concentration was also increased in sEVs-EQ when compared to sEVs-UC 0.25 mL (median [IQR]: 271.2 [260.2–300.3] vs. 34.2 [32.6–35.1] µg/mL, *p* = 0.0058, Figure 1D). The particle to protein ratio was increased in sEVs-EQ compared to sEVs-UC 1 mL, indicating more sEVs and less protein per volume (*p* = 0.0058, Figure 1E) in sEVs-EQ. The RNA concentration was not significantly altered in all groups (Figure 1F). In contrast, the particle to RNA ratio was significantly decreased in all sEVs-UC samples compared to sEVs-EQ, thus indicating a higher number of vesicles and decreased RNA concentration per volume in sEVs-EQ samples.

Isolation with Exoquick resulted in more particles and an equal amount of RNA compared to ultracentrifugation of 0.25 mL plasma. Even though protein amount was increased, we observed CD63, CD 81 and CD9 expression only in sEVs-EQ, and albumin expression was present in sEVs-UC, but not in sEVs-EQ (Figure 1H). Thus, further experiments were performed with sEVs-EQ.

### 2.4. Small Extracellular Vesicles in Cardiac Patients

**Figure 2:**
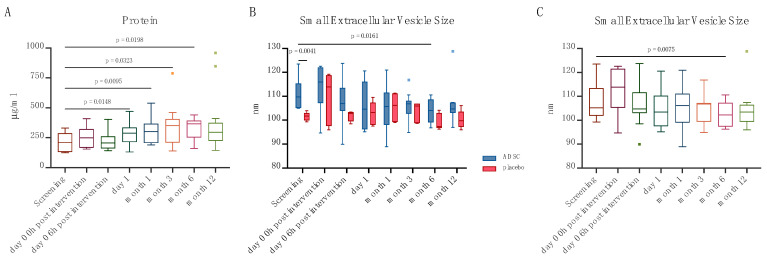
Small EV protein concentration in the overall patient cohort (**A**), small EV size in ADSC and placebo patients (**B**), and the overall patient cohort (**C**).

Neither particle, nor protein, nor RNA concentration were significantly altered by treatment within the follow-up time. Similarly, the particle to protein and particle to RNA ratios did not change. However, in the overall patient cohort, the protein concentration increased significantly at day 1, month 1, month 3 and month 6 (median [IQR]: 244.8 [167.3–287.1] µg/mL vs. 299.9 [214.5–334.4] µg/mL [*p* = 0.0148] vs. 300.6 [207.1–365.9] µg/mL [*p* = 0.0095] vs. 351.6 [223.9–405.3] µg/mL [*p* = 0.0323] vs. 372.6 [277.6–395.6] µg/mL [*p* = 0.0198], Figure 2A).

Small EV size in both groups throughout all timepoints was 105.203 [IQR: 99.680–110.611] nm, which is within the normal range of small EVs. A group difference was only observed at screening, at which point the small EV size was significantly smaller in control patients (median [IQR]: 109.7 [105.2–115.3] nm vs. 101.7 [100.5–101.9] nm, *p* = 0.0041, Figure 2B). In the overall patient cohort, small EV size was reduced at month 6 (median [IQR]: 105.2 [101.9–113.4] nm vs. 102.3 [97.4–107.1] nm, *p* = 0.0075, Figure 2C).

**Figure 3:**
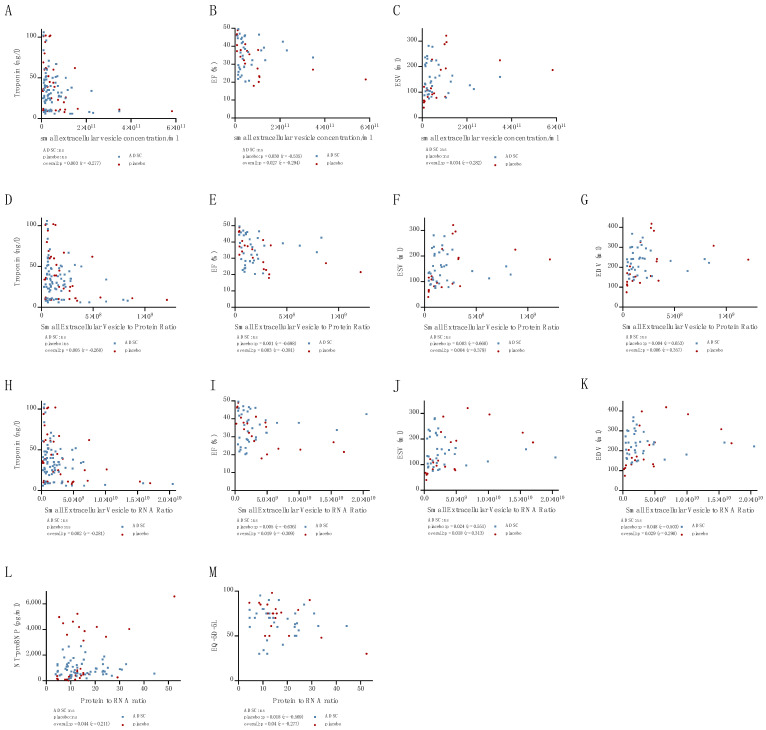
Correlation of small EV concentration/mL with troponin (**A**), EF (**B**), and ESV (**C**). Correlation of small EV to protein ratio with troponin (**D**), EF (**E**), ESV (**F**) and EDV (**G**). Correlation of small EV to RNA ratio with troponin (**H**), EF (**I**), ESV (**J**), EDV (**K**). Correlation of small EV protein to RNA ratio with NT-proBNP (**L**) and EQ-5D-5L (**M**).

Small EV concentration correlated negatively with troponin (*p* = 0.003, r = −0.277, Figure 3A) and ejection fraction (EF) (*p* = 0.027, r = −0.294, Figure 3B), and correlated positively with the end-systolic volume (ESV) (*p* = 0.034, r = 0.282, Figure 3C). The small EV to protein ratio also correlated negatively with troponin (*p* = 0.005, r = −0.260, Figure 3D) and EF (*p* = 0.003, r = −0.381, Figure 3E), and positively with the ESV (*p* = 0.004, r = 0.379, Figure 3F) and end-diastolic volume (EDV) (*p* = 0.006, r = 0.357, Figure 3G). This was also observed for the small EV to RNA ratio: troponin (*p* = 0.002, r = −0.281, Figure 3H), EF (*p* = 0.019, r = −0.309, Figure 2I), ESV (*p* = 0.018, r = 0.313, Figure 3J) and EDV (*p* = 0.029, r = 0.290, Figure 3K).

In contrast, the protein to RNA ratio correlated with NT-proBNP (*p* = 0.044, r = 0.211, Figure 3L) and quality of life EQ-5D-5L score (*p* = 0.04, r = −0.277, Figure 3M).

### 2.5. Small Extracellular Vesicle miRNAs in Cardiac Patients

miR-499a and miR-133a were not present in our samples in any of the sampling timepoints, and miR-1 was only detectable in a handful of samples without data consistency. Thus, those miRNAs were omitted from further analyses.

**Figure 4:**
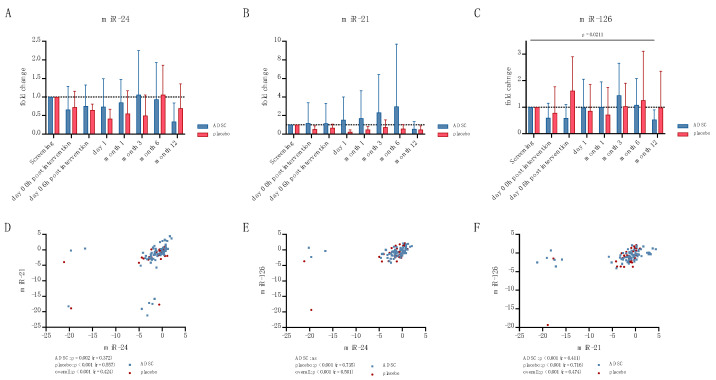
Small EV miR-24 (**A**), miR-21 (**B**), and miR-126 (**C**), and correlations amongst themselves (**D**–**F**).

miR-24 (Figure 4A) and miR-21 (Figure 4B) were both not significantly regulated. However, miR-126 was significantly lower expressed at month 12 in ADSC patients (mean ± SD: 1.00 ± 0.00 vs. 0.528 ± 0.370, *p* = 0.0211, Figure 4C).

Unsurprisingly, the miRNA expression of all three miRNAs correlated positively amongst themselves (miR-24 and miR-21: *p* < 0.001, r = 0.424; miR-24 and miR-126: *p* < 0.001, r = 0.501; miR-21 and miR-126: *p* < 0.001, r = 0.474, Figure 4D–F).

**Figure 5:**
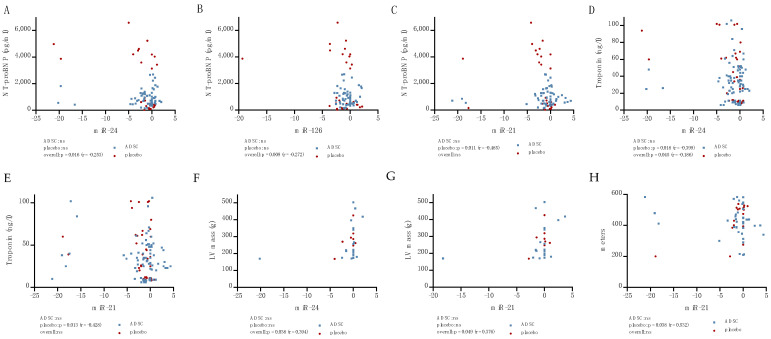
Correlation of NT-proBNP with miR-24 (**A**), miR-126 (**B**), and miR-21 (**C**). Correlation of troponin with miR-24 (**D**) and miR-21 (**E**). Correlation of LV mass with miR-24 (**F**) and miR-21 (**G**) and 6MWT distance with miR-21 (**H**).

Furthermore, a negative correlation of miR-24 (*p* = 0.016, r = −0.253, Figure 5A) and miR-126 (*p* = 0.009, r = −0.272, Figure 5B) with NT-proBNP was observed. In the case of miR-21, this correlation was only observed in placebo patients (*p* = 0.011, r = −0.485, Figure 5C). miR-24 expression and troponin also showed a negative correlation (*p* = 0.045, r = −0.186, Figure 5D) whilst, for miR-21, this could again only be observed in placebo patients (*p* = 0.013, r = −0.428, Figure 5E).

No significant correlation of small EV miRNAs with the EF, ESV or EDV was observed. However, an increasing miR-24 (*p* = 0.0038, r = 0.394, Figure 5F) and miR-21 (*p* = 0.049, r = 0.376, Figure 5G) expression correlated with an increased LV mass. An increased miR-21 expression in control patients was also associated with an increased walking distance in the 6 min walking test (6MWT) (*p* = 0.038, r = 0.532, Figure 5H).

Even though all three miRNAs are mostly known for their cardiac regulatory potential, we observed a negative correlation with GGT, LDH, and CRP, and a positive correlation with eGFR (supplementary data).

For all three miRNAs, a negative correlation with lipid metabolism parameters were observed, but this finding was only significant for the overall patient cohort for miR-126 and cholesterol (*p* = 0.003, r = −0.328, supplementary data). For HDL, LDL, and cholesterol, this correlation was only observed in the placebo group (supplementary data). In contrast, lipoprotein (a) positively correlated with all three miRNAs in the ADSC group (supplementary data).

## 3. Discussion

This is the first study investigating circulating small EVs and their miRNA cargo in patients with ischemic heart disease treated with percutaneously intramyocardially administered allogeneic ADSCs.

The patient collective described within our analyses were classified as untreatable with conventional medical interventions, as both their clinical presentation and cardiac parameters indicated the presence of significant heart failure despite the highest degree of feasible optimal pharmacological management, whilst being deemed unlikely to benefit from further interventions or surgery. Fortunately, only a small number of patients meet this criterium. Nonetheless, it is precisely this patient cohort that is in desperate need of novel regenerative therapies. In the SCIENCE study, the concept of allogeneic ADSCs derived from healthy donors was studied as an off-the-shelf product alternative with the potential to offer an easy, comparably quick, and standardized regenerative cell-based therapy. Despite the high expectations, and the fact that the SCIENCE study once again managed to confirm the safety aspect of cell-based regenerative therapy, it did not report an effect on the primary endpoint of improved cardiac function 6 months after treatment (assessed by left ventricular ESV in echocardiography) [32].

The reasons for the mostly unimpressive results of cell-based regenerative therapies have long been a topic of discussion. Whilst not yet clearly elucidated, the lack of substantial benefits of these therapies might be at least partially attributable to the low long-term cell engraftment, which has previously been reported as only 5% after 2 h, and 1% after 20 h. Thus, the limited survival of transplanted cells has often been discussed [33]. Furthermore, cardiac transdifferentiation of transplanted cells to support cardiac regeneration in acute myocardial infarction was not observed in vivo [34]. Nevertheless, several studies still described moderate positive effects of cell-based regenerative therapies, indicating the involvement of other effector mechanisms. The mode of action of these non-cell-mediated effects of cell-based regenerative therapies is far from being understood [35,36]. It was, however, demonstrated that the paracrine effects are at least partly mediated through EVs, and that administering MSC derived small EVs might also result in cardioprotective effects [37].

The aim of this subanalysis was to investigate the effect of ADSC treatment on small EVs and their miRNA cargo. In the light of the lack of measurable effects of ADSC treatment on functional indicators or quality of life in this study, the only minor effects on small EV levels do not come as a surprise. While miR-126 was significantly reduced in ADSC patients after 12 months, no other group differences were observed. Furthermore miR-24 and miR-126 showed a significant negative correlation with NT-proBNP, while miR-21 and miR-24 further negatively correlated with troponin. Additionally, small EV miRNAs also correlated with liver function, inflammation, and lipid metabolism parameters.

EVs have emerged as a subject of interest within the past years, and a myriad of data have been published on this topic. However, despite the extensive effort made in clarifying definitions in EV research, upon studying the currently available literature, one is quickly confronted with varying and mostly un- or vaguely defined EV subclasses. This makes comparing or interpretating previously published findings, as well as establishing the novelty and reproducibility of newly obtained results, difficult. In its most recent position statement published in 2018, the International Society for Extracellular Vesicles (ISEV) has simplified the nomenclature [10] on the basis of the observation that, in the light of the current widespread isolation and characterization methods, no certain assertion regarding EV origin can be made. Although EVs are an interesting, and by now omnipresent, research field, no gold standard for the isolation and characterization of EVs has been established thus far, resulting in the application of a number of methods with varying rates of success. The choice of method also has a pivotal impact on the biological cargo profile, including miRNAs. Ultracentrifugation is an uncomplicated method for the isolation of EVs that generally results in a respectable yield with good quality. Therefore, it is the method of choice of many researchers. Nevertheless, various protocols are being performed, emphasizing a fortiori inconsistencies in isolation methods [38]. 

EV RNA profiles generally reflect the transcriptome of their cells of origin. However, some distinct differences can be observed, suggesting selective packing of miRNAs in small EVs [39,40]. Small EV miRNAs vary in different cell types and diseases [41,42]. Thus, we hypothesized that the percutaneous administration of allogeneic ADSCs in ischemic heart disease patients might be reflected in small EVs isolated from patient plasma, and that detecting such alterations might offer potential insights into the interpretation of the modes of action of cell-based regeneration therapies, which might not have been accounted for thus far. In ischemic heart disease, the myocardium and kidney have been proposed as the main source of circulating EVs [5].

We chose six cardiac miRNAs as the focus of our investigation. Of those, only three were expressed in plasma small EVs in our patients. Despite being previously described as cardiac miRNAs with systemic expression patterns, miR-1, miR-133a and miR-499 were not (sufficiently) expressed in our samples, and were thus not included in further analyses [18,19,21]. Circulating small EV miRNAs are not only of interest as biomarkers, but might also enable inferences concerning cardiac miRNA expression. In a rat model of heart failure with preserved EF, the plasma levels of small EV miR-126 were decreased, and correlated with myocardial expression levels [20]. In contrast, the serum small EV miR-126 was increased in patients with acute myocardial infarction, and miR-21 was decreased in the same population [43]. Similarly to severe cardiac ischemia, remote ischemic preconditioning also resulted in induced serum small EV miR-126 expression [44]. The same effect was also observed for plasma small EV miR-24 [45]. Serum small EV miR-21 has also been found to be significantly increased in heart failure patients [46]. Furthermore, serum small EV miR-21 can also be used to diagnose cardiac sarcoidosis [47]. Both miR-126 and miR-24 in plasma small EVs can be used to distinguish the severely dilated aorta in bicuspid aortic valves from a less dilated aorta [48].

Several studies have attempted to confirm and harness the regenerative potential of the above-mentioned miRNAs delivered as miR-loaded small EVs in in vitro and small animal studies. miR-126 is crucially involved in regulating vascular integrity and angiogenesis [22]. miR-126- and miR-146a-loaded small EVs in alginate derivative hydrogels were, in fact, able to reduce infarct size and fibrosis, and increase angiogenesis, in a rat myocardial infarction model [49]. miR-21 is a cardioprotective miRNA, which prevents oxidative stress-related apoptosis in ischemic cardiomyocytes. Serum EVs containing miR-21 reduced cardiomyocyte apoptosis in vitro and in vivo through inhibition of programmed cell death 4 (PCD4) and reduction in Bax/Bcl-2 [23]. In contrast, ischemic cardiomyocytes take up miR-21*-containing small EVs released from cardiac fibroblasts, which results in hypertrophy and fibrosis [40]. Another cardioprotective effect of miR-21 is the activation of angiogenesis. Small EV miR-21 was significantly dysregulated in cardiomyocytes derived from failing hearts, and its administration was linked to impaired endothelial tube formation [42]. The cardiac miRNA miR-24 is upregulated in cardiac endothelial cells as a response to ischemia, and induces endothelial cell apoptosis and inhibits angiogenesis [50]. In contrast, miR-24 displays antiapoptotic effects in cardiomyocytes and attenuates cardiac fibrosis [51,52].

With regard to the interpretation of the results of our study, several limitations must be mentioned. Firstly, fifteen patients is a prohibitively small size, especially considering that only five patients received the placebo, due to the randomization protocol, thus offering the additional challenge of unbalanced group sizes to an already small initial cohort (2:1 randomization of ADSCs and placebo). We are aware that such a small sample size can only generate hypotheses. The very strong inclusion and exclusion criteria allowed us to include a limited number of patients in a limited time frame, even though many more patients were reviewed. In addition, due to well-balanced patient characteristics between the centers in the multicenter study, a pre-defined number of patients per center was defined. Additionally, in the light of the non-existent clinical effect of the ADSC treatment itself, and the primary hypothesis postulating that the observed small EV (cargo) changes would be linked to the treatment success, the lack of any observable significant differences between ADSC and placebo patients is in line with our predictions. However, small EV miRNAs are known to point towards subclinical effects, and those might have simply been missed as a result of the small sample size. Furthermore, only men were included in this study. Even though female patients were contacted, none were willing to participate in this study. Due to the well-established relevance of gender-specific differences in disease presentation and therapy response, a possible treatment effect might have been missed due to low inclusion rates of women, a consideration not only relevant for this study, but the area of regenerative cardiac therapy in general.

## 4. Materials and Methods

### 4.1. Study Design

This SCIENCE substudy reports the analysis of plasma small EV cardiac miRNA expression of patients included in the Vienna center. The study was approved by the EU Commission (Grant Agreement 643478), the local ethics committee of the Medical University of Vienna (EC number: 1868/2015), and national regulatory authorities. It was conducted according to the declaration of Helsinki, and all patients gave written informed consent before enrolment.

### 4.2. Patients

All patients participated in the European multicenter, phase II, double blinded, placebo-controlled SCIENCE trial, and were treated at the Medical University of Vienna. [32] Fifteen patients with non-treatable symptomatic heart failure with reduced EF (HFrEF) were included in the Vienna center, and randomized to receive either allogeneic ADSC (group ADSC) or sodium chloride (group placebo). Patients were 2:1 randomized, thus ten patients received ADSCs and five patients received the placebo.

**Figure 6:**
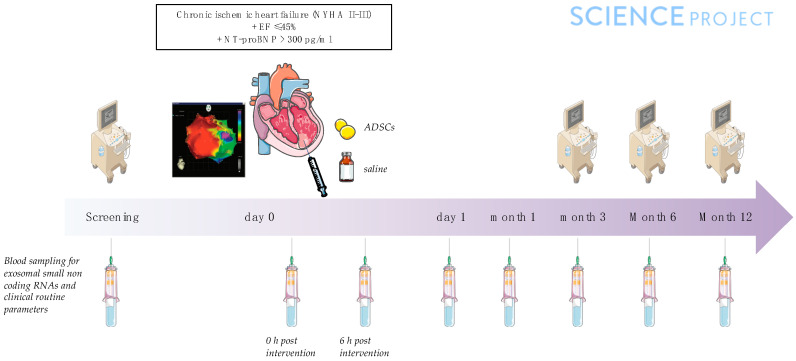
Study flow.

Major inclusion criteria were left ventricular EF (LVEF) < 45%, NT-proBNP > 300 pg/mL and NYHA class II-III at screening. Details of the rationale, design, and detailed inclusion and exclusion criteria of the SCIENCE trial can be found in the 2019 published article by Paitazoglou et al. (Figure 6) [53].

The primary endpoint of this study was improvement of LVESV in echocardiography 6 months after treatment.

### 4.3. Blood Sampling

Peripheral venous blood for either routine testing or small EV isolation was drawn at screening, day 0 at both 0 h and 6 h post-intervention, day 1, month 1, month 3, month 6, and month 12. Blood for routine testing was brought to the Department of Laboratory Medicine at the Medical University of Vienna, and measured according to current standards. Apart from the day of intervention at 0 h and 6 h post-intervention, regular blood testing included blood count, coagulation, kidney function, liver function, electrolytes, lipids, cardiac and inflammation parameters. At the intervention day, only cardiac biomarkers were evaluated. eGFR was calculated with the CKD-EPI 2021 formula [54].

**Figure 7:**
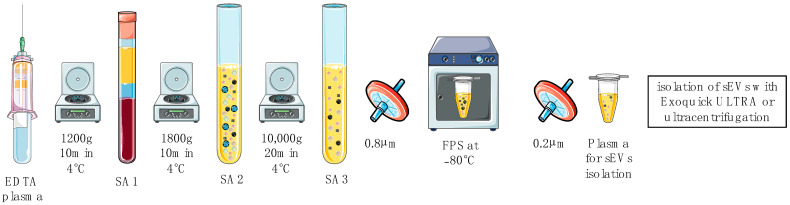
Overview of plasma preparation for small EV isolation.

For small EV isolation, EDTA plasma was obtained and subjected to sequential centrifugation prior to the isolation (Figure 7). Firstly, EDTA blood was centrifuged at 1200× *g* for 10 min at 4 °C; secondly, the supernatant (SA1) was transferred to a new tube and another centrifugation step at 1800× *g* for 10 min at 4 °C was performed. The last centrifugation step was performed after transferring the supernatant (SA2) to new vials at 10,000× *g* for 20 min at 4 °C. Then, the supernatant (SA3) was filtered through 0.8 μm surfactant-free cellulose acetate filters (16592, Sartorius, Goettingen, GER). Final plasma samples (FPS) were stored at −80 °C until further processing. The pellet after the last centrifugation step was defined as microvesicles (MVs) for further experiments.

### 4.4. Small Extracellular Vesicle Isolation

Two different methods for small EV isolation were performed as preliminary tests to identify the most effective and efficient method for small EV isolation.

#### 4.4.1. Ultracentrifugation

After thawing the sequentially centrifuged plasma (FPS), it was filtered through 0.2 μm surfactant-free cellulose acetate filters (16534, Sartorius, Goettingen, GER). In order to test different starting amounts, samples of 0.25, 0.5 and 1 mL of plasma with phosphate-buffered saline without calcium/magnesium (PBS–/–), to add up to 10 mL, were centrifuged at 120,000× *g* at 4 °C for 2 h in thinwall polypropylene tubes in an ultracentrifuge with a swing out rotor (SW 41 TI). The supernatant was discarded, and the pellet was resuspended in 10 mL PBS–/–. A second round of ultracentrifugation with equal settings was performed. The supernatant was discarded again, and the pellet (sEVs-UC 1 mL/0.5 mL/0.25 mL) was resuspended in 100 μL PBS–/– and stored at −80 °C until further use.

#### 4.4.2. Exoquick ULTRA EV Isolation Kit

Small EV isolation with the Exoquick ULTRA EV Isolation Kit for Serum and Plasma (EQULTRA-20A-1, System Biosciences, Palo Alto, CA, USA) was performed according to the manufacturer’s protocol. In brief, sequentially centrifuged plasma (FPS) was filtered through 0.2 μm surfactant-free cellulose acetate filters (16534, Sartorius, Goettingen, GER), 250 μL of plasma was used as sample volume, and 67 μL ExoQuick was added, mixed well by inverting, and incubated at 4 °C for 30 min. Centrifugation of the plasma-ExoQuick samples at 4 °C and 3000× *g* for 10 min was performed, and EVs appeared as a pellet. Supernatant was discarded, and 200 μL each of Buffer B and Buffer A were added to the pellet. Several centrifugation steps through the column were performed to prepare it for loading with small EVs. After loading, the column with small EVs incubation for 5 min with rotation was performed. A centrifugation step at 1000× *g* for 30 s was performed to collect small EVs in a tube (sEVs-EQ), the column was then discarded.

### 4.5. Characterization of Small Extracellular Vesicles with Nanoparticle Tracking Analysis

Size and concentration of small EVs were characterized by nano-particle tracking analysis (NTA). Samples containing small EVs were diluted with fresh sterile PBS–/– to a concentration allowing optimal measurements. Diluted samples were analyzed using the ZetaVIEW device from Particle Metrix (ZetaVIEW S/N 17-323, Inning am Ammersee, GER; Camera 0.743 μm/px; Software ZetaView 8.04.02). Prior to each measurement session, daily performance was assessed with polystyrene size standard beads PS100 at a known concentration and size. Measurements were performed at the following settings: 24 °C, sensitivity 70, shutter 100, minimal brightness 25, minimal area 6, maximum area 5000. After injection of the sample into the chamber, it was allowed to equilibrate for 4 min before the measurement (each three rounds at eleven positions). All valid measurements per sample were averaged.

### 4.6. Characterization of Small Extracellular Vesicles with Coomassie Assay

Coomassie assay was used for detection of protein amounts in small EVs. As standard, a serial dilution of 25 μg/mL to 2.5 μg/mL bovine serum albumin (A3608, Sigma-Aldrich, St. Luis, MO, USA) in PBS–/– was used. Each 30 μL of sample/standard and Coomassie assay reagent (23200, Thermo Fisher Scientific, Waltham, MA, USA) were added to wells in a 384 well plate (781061, Greiner Bio-One, Kremsmuenster, AUT) and gently mixed for 30 s at a plate shaker. After an incubation period of 10 min at room temperature in dark conditions, absorbance was measured at 595 nm using a TECAN SPARK 20M (TECAN, Morrisville, NC, USA). AutoOptical density values of samples were compared to the standard curve generated with known concentrations of protein. 

### 4.7. Characterization of Small Extracellular Vesicles with Western Blot

Western blot was performed to characterize the expression of small EV-associated antigens in small EVs isolated with ultracentrifugation and Exoquick, in comparison to MVs and plasma. Samples (1 μg) were mixed with Laemmli buffer (with β-mercaptoethanol for albumin and without β-mercaptoethanol for CD9, CD63 and CD81) and boiled at 90 °C for 10 min to denature and reduce the samples. Afterwards samples and a prestained protein ladder as reference (26616, Thermo Fisher Scientific, Waltham, MA, USA) were applied to the wells of a 10% BOLT bis-tris plus gel (NW00102BOX, Thermo Fisher Scientific, Waltham, MA, USA) and electrophoresis was performed in an XCell SureLock Mini-Cell Electrophoresis System (Thermo Fisher Scientific, Waltham, MA, USA) with 1× MOPS SDS running buffer (13226499, Thermo Fisher Scientific, Waltham, MA, USA) at 200 V, 90 W, 530 mA for 45 min. After electrophoresis, the gel was allowed to equilibrate in 1× BOLT transfer buffer (BT0006, Thermo Fisher Scientific, Waltham, MA, USA) with 20% methanol (10284580, Fisher Scientific, Schwerte, GER) for 10 min. Protein transfer onto 0.2 μm PVDF membranes (1620177, Bio-Rad Laboratories, Hercules, CA, USA) was performed in BOLT transfer buffer with 20% methanol at 30 V, 100 mA, 90 W for 16 h. To assess successful transfer, the membrane was stained with the Revert 700 total protein stain kit (926-11010, LI-COR Biosciences, Lincoln, NE, USA) and the gel was stained with SimplyBlue SafeStain (LC6060, Thermo Fisher Scientific, Waltham, MA, USA). As only 1 μg of protein was available for Western blot analysis, SuperSignal™ Western Blot Enhancer (46640, Thermo Fisher Scientific, Waltham, MA, USA) was used according to the manufacturer’s protocol. Blocking of the membrane was performed in 5% milk in 1× TBST for 1 h at room temperature. Incubation with primary antibodies was performed overnight. A list of used primary antibodies, as well as dilutions, can be found in Supplementary Table S1. Incubation with secondary antibody (ab6721, Abcam, Cambridge, GBR) in 2% milk in TBST was performed for 1 h at room temperature. Chemiluminescence visualization was performed with Radiance Plus (AC2103, Azure Biosystems, Dublin, CA, USA) at an Azure c600 (Azure Biosystems, Dublin, CA, USA).

### 4.8. RNA Isolation, cDNA Synthesis and qPCR

RNA isolation of plasma small EVs was performed using the miRNeasy Serum/Plasma kit (217184, Qiagen, Venlo, NLD). Briefly, 1 μL of spike-in (UniSp2, UniSp4, UniSp5; 339390, Qiagen, Venlo, NLD) was added to 1000 μL of Qiazol lysis reagent (79306, Qiagen, Venlo, NLD). Then, 200 μL of the small EV sample was added. After vortexing and incubation for 5 min, 200 μL chloroform was added, vortexed, and incubated again. After a centrifugation step (12,000× *g* at 4 °C for 15 min), the aqueous phase was transferred to a new tube, mixed with 100% ethanol, and transferred on a spin column. Several steps of centrifugation were performed until final elution in 12 μL RNAse-free H_2_O. Prior to storage at −80 °C, RNA amount and quality was measured with a Nanodrop spectrophotometer (Thermo Fisher Scientific, Waltham, MA, USA).

cDNA synthesis was performed using the miRCURY LNA RT Kit (339340, Qiagen, Venlo, NLD) according to the manufacturer’s protocol. In brief, 5.5 μL RNA was mixed with 4.5 μL mastermix, including UniSp6/CelmiR39 spike in control and 12U heparinase I (P0735L, New England Biolabs, Ipswich, MA, USA), as patients received heparin during the intervention. After incubation for 60 min at 42 °C, and 5 min at 95 °C, it was resuspended in 90 μL RNAse-free H_2_O and stored at −20 °C.

miRNA qPCR was performed with the miRCURY LNA SYBR Green PCR kit (339345, Qiagen, Venlo, NLD) according to the manufacturer’s protocol on an Applied Biosystems 7500 Real-Time PCR System (Life Technologies, Carlsbad, CA, USA). A list of miCURY miRNA primer assays can be found in Supplementary Table S2. The relative gene expression level was calculated using the ΔΔCt method (i.e., expression levels relative to expression of UniSp2/6 and to an endogenous control). The expression changes were calculated as relative to expression to baseline/screening per patient.

### 4.9. Statistics

Data obtained were evaluated using IBM SPSS Statistics version 28 (SPSS Inc., Chicago, IL, USA) and GraphPad Prims 9 software (GraphPad Software Inc., LA Jolla, CA, USA). Graphs were made with GraphPad Prims 9 software and R version 4.1.3 using ggplot2 version 3.3.6. Normal distribution was tested using Kolmogorov–Smirnov test. Parametric variables were expressed as mean ± standard deviation (SD) and non-parametric variables were expressed as median and interquartile range (IQR). A mixed-effects model was calculated for repeated measurements, to compare time-dependent and treatment-dependent effect. Dunnett’s multiple comparisons test was applied to correct for multiple testing. A Pearson correlation test was performed for parametric variables, and a Spearman rank test for non-parametric variables. All tests were performed in a two-sided manner, and *p*-values below 0.05 were considered statistically significant.

## Data Availability

Pseudonymised participant data will be made available upon qualified request starting with publication. Approval of a proposal and a signed data access agreement are mandatory.

## Supplementary Materials

The following supporting information can be downloaded at: https://www.mdpi.com/article/10.3390/ijms241310647/s1.
