# Supplementary material for: Plasma Small Extracellular Vesicle Cardiac miRNA Expression in Patients with Ischemic Heart Failure, Randomized to Percutaneous Intramyocardial Treatment of Adipose Derived Stem Cells or Placebo: Subanalysis of the SCIENCE Study
Source: Int J Mol Sci. 2023 Jun 26;24(13):10647. doi: 10.3390/ijms241310647 (PMC10342130; doi:10.3390/ijms241310647)
Supplement: Supplementary file 1 [file ijms-24-10647-s001.zip › ijms-2438188-supplementary.pdf]

Supplementary Material to

Plasma Small Extracellular Vesicle Cardiac miRNA Expression in Patients with Ischemic Heart Failure, Randomized to Percutaneous Intramyocardial Treatment of Adipose Derived Stem Cells or Placebo: Subanalysis of the SCIENCE Study

Supplementary Table S1: List of primary antibodies for western blot analysis

	Dilution	Article number	Company
Primary antibodies			
Albumin	1:1000	4929S	Cell Signalling Technology
CD9	1:500	EXOAB-CD9A-1	System Biosciences
CD63	1:1000	40192	Signalling Antibody
CD81	1:1000	41779	Signalling Antibody
Secondary antibody			
Goat anti-rabbit	1:10 000	ab6721	Abcam

Supplementary Table S2: List of miRCURY miRNA Primer Assays

**Name**	**Article number**	**Company**
UniSp2	YP00203950	Qiagen
UniSp6	YP00203954	Qiagen
hsa-miR-1-3p	YP00204344	Qiagen
hsa-miR-24-3p	YP00204260	Qiagen
hsa-miR-133-3p	YP00204788	Qiagen
hsa-miR-21-5p	YP00204230	Qiagen
hsa-miR499a-5p	YP00205935	Qiagen
hsa-miR-126-3p	YP00204277	Qiagen

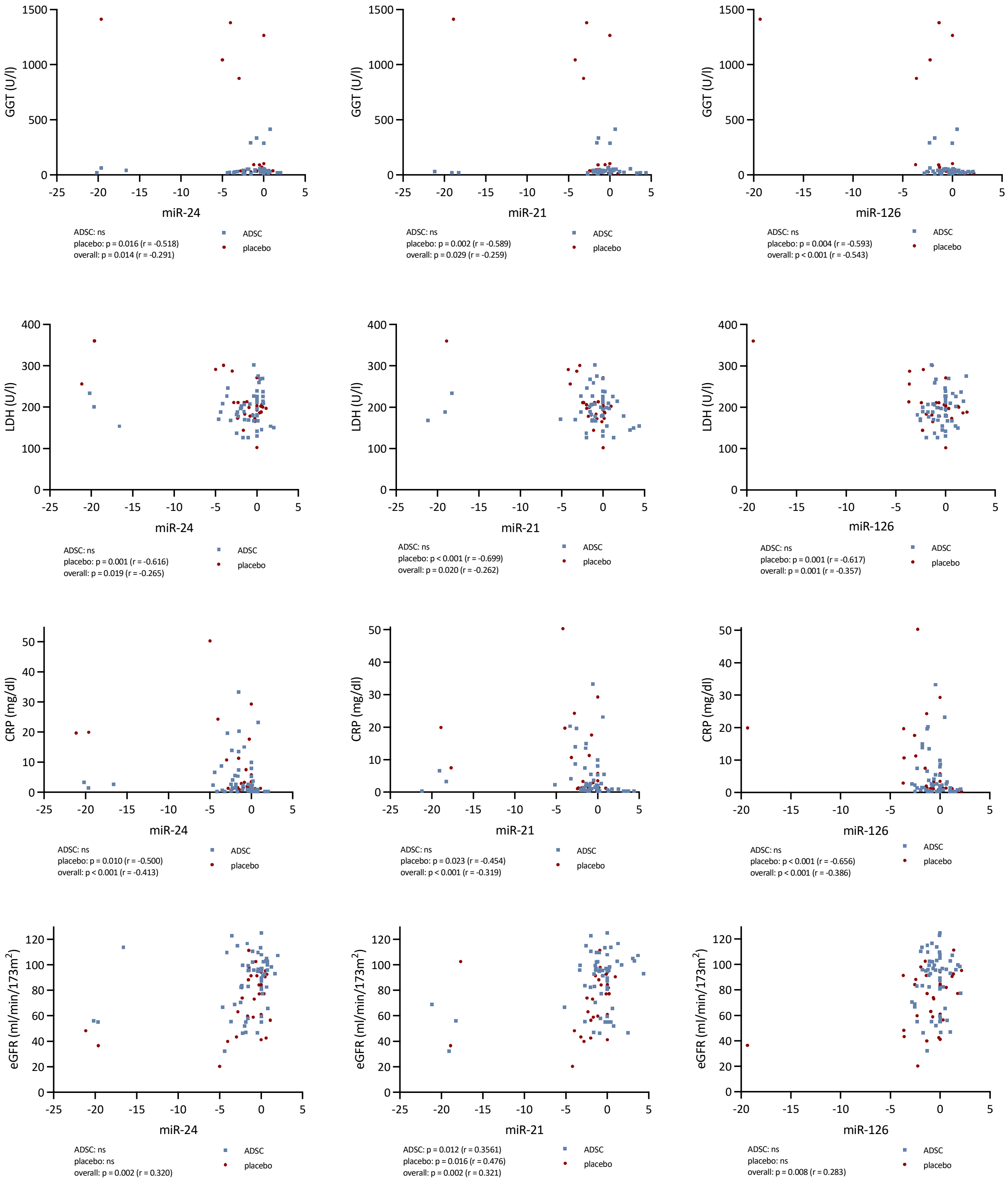

Supplementary Figure S1:Correlation of small EV miRNAs with GGT, LDH, CRP and eGFR

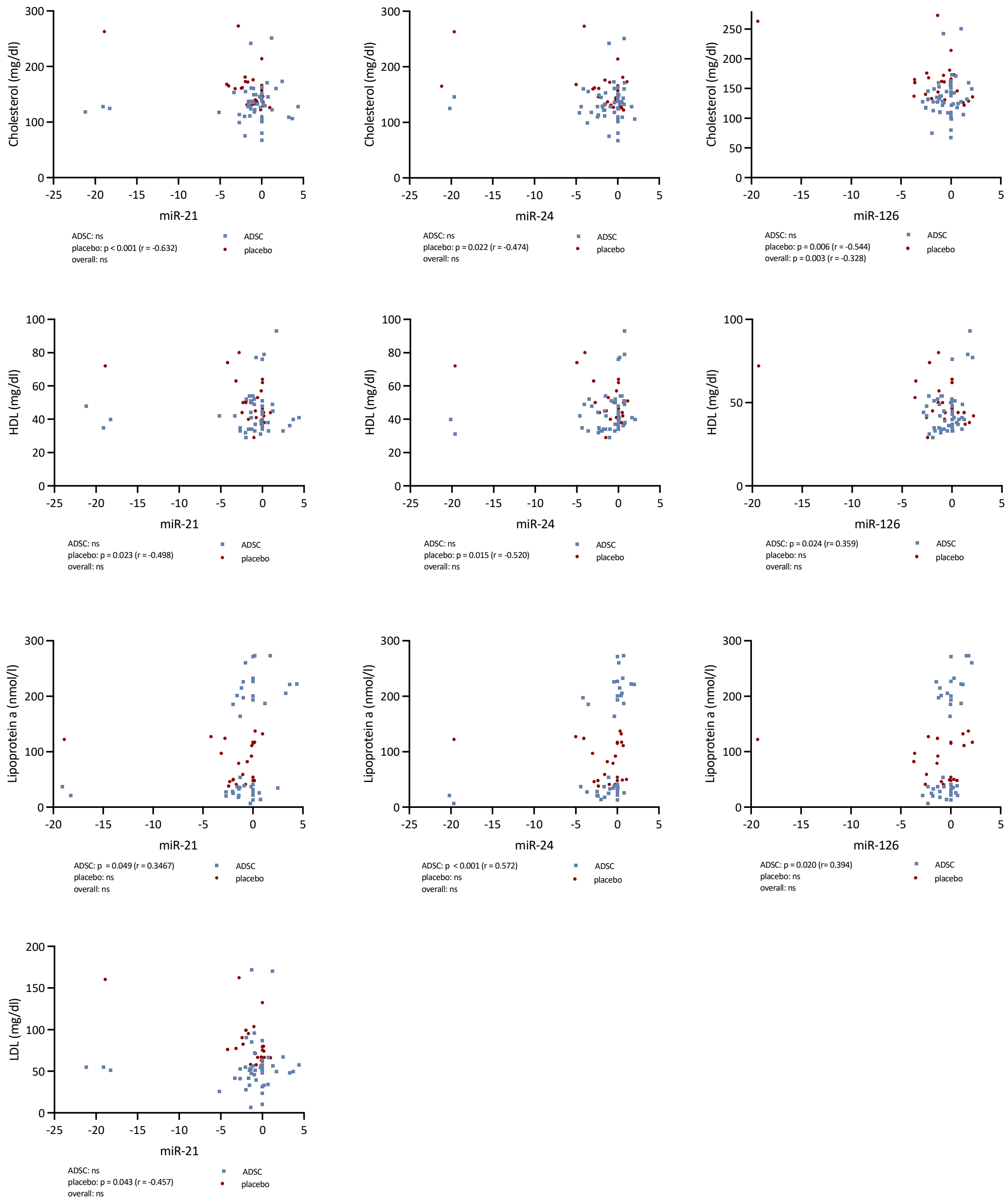

Supplementary Figure S2: Correlation of small EV miRNAs with lipid metabolism parameters
